# 2-(2-Eth­oxy-2-oxoacetamido)­benzoic acid

**DOI:** 10.1107/S2414314620006033

**Published:** 2020-05-06

**Authors:** MingChao Yu, LinLin Wang, LingYang Wang, ZhiYong Wu

**Affiliations:** a School of Medicine and Pharmacy and College of Marine Life Science, Ocean University of China, Qingdao, Shandong 266003, People’s Republic of China; Zhejiang University (Yuquan Campus), China

**Keywords:** crystal structure, oxamide derivative, hydrogen bonds

## Abstract

The title compound, C_11_H_11_NO_5_, has a nearly planar geometry. In the crystal, the mol­ecules are assembled into chains parallel to the [  11] direction by O—H⋯O and C—H⋯O hydrogen bonds.

## Abstract 2

The title compound has a nearly planar geometry. In the crystal, the mol­ecules are assembled into chains parallel to the [ 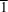 11] direction by O—H⋯O and C—H⋯O hydrogen bonds

## Graphical abstract

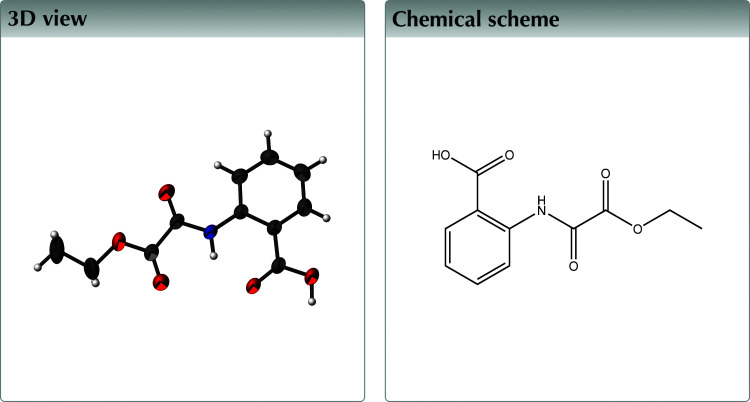

## Structure description

Oxamide derivatives are of considerable current inter­est because of their DNA-binding properties and cytotoxic activity (Martínez-Martínez *et al.*, 1998 ▸; Li *et al.*, 2012 ▸; Yue *et al.*, 2012 ▸; Zheng *et al.*, 2012 ▸). As part of our studies in this area, we report the structure of the title compound herein.

**Figure 1:**
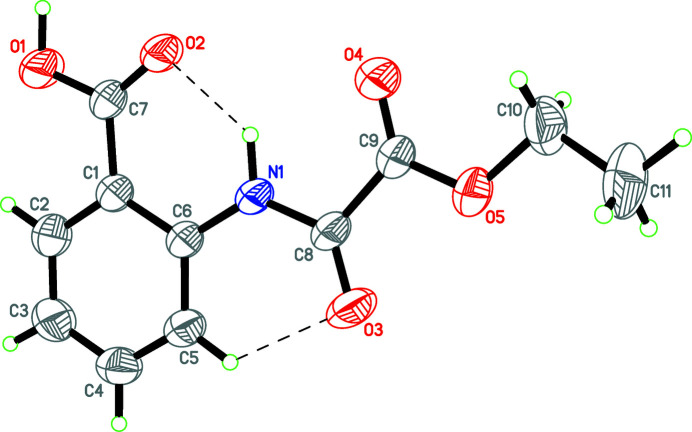
Mol­ecular structure of the title compound with the atom labelling. Displacement ellipsoids are drawn at the 50% probability level. Hydrogen bonds (Table 1 ▸) are indicated by dashed lines.

The asymmetric unit contains one title compound adopting the expected *transoid* conformation (Fig. 1 ▸). The mol­ecule has an almost planar geometry except for the terminal methyl group; atom C11 deviates by 0.388 (3) Å from the mean plane of other atoms. Intra­molecular N—H⋯O and C—H⋯O hydrogen bonds occur (Table 1 ▸).

**Figure 2:**
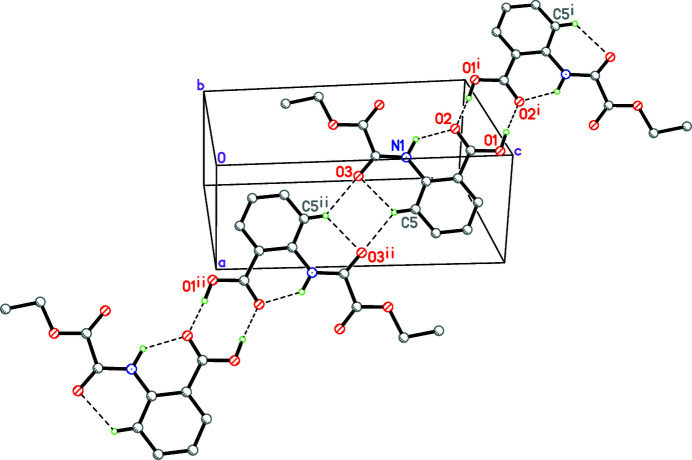
A one-dimensional hydrogen-bonded chain along [ 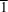 11]. Hydrogen bonds (Table 1 ▸) are indicated by dashed lines. H atoms not involved in hydrogen bonding are omitted for clarity. Symmetry codes: (i) −*x*, 1 − *y*, 2 − *z*; (ii) 1 − *x*, −*y*, 1 − *z*.

In crystal, pairs of mol­ecules are linked by O—H⋯O hydrogen bonds (Table 1 ▸) into inversion dimers characterized by an 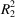 (8) motif (Fig. 2 ▸). The dimers are linked by further C—H⋯O hydrogen bonds with an 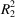 (12) motif, giving rise to a chain extending along the [ 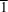 11] direction.

## Synthesis and crystallization

The title compound was synthesized using a literature method (Matović, 2005 ▸). Ethyl chloro­oxo­acetate (10 mmol, 1.13 ml) in 10 ml of tetra­hydro­furan (THF) was added dropwise to a 10 ml THF solution containing 2-amino­benzoic acid (10 mmol, 1.37 g) at 273 K. The resulting solution was stirred for 2 h. The solution was concentrated under vacuum and the compound was precipitated as a yellowish powder then washed with ether and dried under vacuum. Well-shaped yellowish single crystals were obtained by slow evaporation of an ethanol solution of the recrystallized product. Yield: 55%. Analysis calculated for C_11_H_11_NO_5_: C, 55.70; H, 4.67; N, 5.90%. Found: C, 55.77; H, 4.64; N, 5.92%.

## Refinement

Crystal data, data collection and structure refinement details are summarized in Table 2 ▸.

## Supplementary Material

Crystal structure: contains datablock(s) I. DOI: 10.1107/S2414314620006033/xu4040sup1.cif

Structure factors: contains datablock(s) I. DOI: 10.1107/S2414314620006033/xu4040Isup3.hkl

Supporting information file. DOI: 10.1107/S2414314620006033/xu4040Isup3.cml

CCDC reference: 2000864

Additional supporting information:  crystallographic information; 3D view; checkCIF report

## Figures and Tables

**Table 1:** Hydrogen-bond geometry (Å, °)

*D*—H⋯*A*	*D*—H	H⋯*A*	*D*⋯*A*	*D*—H⋯*A*
O1—H1⋯O2^i^	0.96 (3)	1.67 (3)	2.6274 (16)	176 (2)
N1—H1*A*⋯O2	0.93 (2)	1.92 (2)	2.6574 (15)	135.1 (16)
C5—H5⋯O3	0.948 (19)	2.230 (18)	2.866 (2)	123.7 (14)
C5—H5⋯O3^ii^	0.948 (19)	2.36 (2)	3.1107 (19)	135.9 (14)

Symmetry codes: (i) 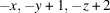 ; (ii) 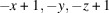 .

**Table 2:** Experimental details

Crystal data	
Chemical formula	C_11_H_11_NO_5_
*M*_r_	237.21
Crystal system, space group	Triclinic, *P* 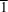
Temperature (K)	296
*a*, *b*, *c* (Å)	4.8774 (13), 9.470 (3), 12.719 (3)
α, β, γ (°)	106.784 (7), 97.222 (7), 92.444 (8)
*V* (Å^3^)	556.1 (3)
*Z*	2
Radiation type	Mo *K*α
μ (mm^−1^)	0.11
Crystal size (mm)	0.73 × 0.24 × 0.19

Data collection	
Diffractometer	Bruker *APEX* area detector
Absorption correction	Multi-scan (*SADABS*; Bruker, 2002 ▸)
No. of measured, independent and observed [*I* > 2σ(*I*)] reflections	8969, 2557, 2016
*R*_int_	0.028
(sin θ/λ)_max_ (Å^−1^)	0.653

Refinement	
*R*[*F* ^2^ > 2σ(*F* ^2^)], *wR*(*F* ^2^), *S*	0.049, 0.138, 1.06
No. of reflections	2557
No. of parameters	198
H-atom treatment	All H-atom parameters refined
Δρ_max_, Δρ_min_ (e Å^−3^)	0.24, −0.27

Computer programs: *SMART* and *SAINT* (Bruker, 2002 ▸), *XP* in *SHELXTL* and *SHELXS97* (Sheldrick, 2008 ▸), *SHELXL2014/7* (Sheldrick, 2015 ▸) and *publCIF* (Westrip, 2010).

## full crystallographic data

### Crystal data

**Table:**

C_11_H_11_NO_5_	*Z* = 2
*M**_r_* = 237.21	*F*(000) = 248
Triclinic, *P*1	*D*_x_ = 1.417 Mg m^−^^3^
*a* = 4.8774 (13) Å	Mo *K*α radiation, λ = 0.71073 Å
*b* = 9.470 (3) Å	Cell parameters from 4983 reflections
*c* = 12.719 (3) Å	θ = 2.4–27.7°
α = 106.784 (7)°	µ = 0.11 mm^−^^1^
β = 97.222 (7)°	*T* = 296 K
γ = 92.444 (8)°	Plate, light yellow
*V* = 556.1 (3) Å^3^	0.73 × 0.24 × 0.19 mm

### Data collection

**Table:**

Bruker APEX area detector diffractometer	2016 reflections with *I* > 2σ(*I*)
Radiation source: fine-focus sealed tube	*R*_int_ = 0.028
ω scans	θ_max_ = 27.7°, θ_min_ = 3.2°
Absorption correction: multi-scan (SADABS; Bruker, 2002)	*h* = −6→6
	*k* = −12→12
8969 measured reflections	*l* = −16→16
2557 independent reflections	

### Refinement

**Table:**

Refinement on *F*^2^	0 restraints
Least-squares matrix: full	Hydrogen site location: difference Fourier map
*R*[*F*^2^ > 2σ(*F*^2^)] = 0.049	All H-atom parameters refined
*wR*(*F*^2^) = 0.138	*w* = 1/[σ^2^(*F*_o_^2^) + (0.0728*P*)^2^ + 0.0917*P*] where *P* = (*F*_o_^2^ + 2*F*_c_^2^)/3
*S* = 1.06	(Δ/σ)_max_ < 0.001
2557 reflections	Δρ_max_ = 0.24 e Å^−^^3^
198 parameters	Δρ_min_ = −0.27 e Å^−^^3^

### Special details

**Table:**

Geometry. All esds (except the esd in the dihedral angle between two l.s. planes) are estimated using the full covariance matrix. The cell esds are taken into account individually in the estimation of esds in distances, angles and torsion angles; correlations between esds in cell parameters are only used when they are defined by crystal symmetry. An approximate (isotropic) treatment of cell esds is used for estimating esds involving l.s. planes.
Refinement. All H atoms were found in a difference Fourier map and refined freely.

### Fractional atomic coordinates and isotropic or equivalent isotropic displacement parameters (Å^2^)

**Table:**

	*x*	*y*	*z*	*U*_iso_*/*U*_eq_	
O1	0.2576 (3)	0.38020 (16)	1.03091 (9)	0.0681 (4)	
O2	0.0551 (2)	0.41447 (12)	0.87522 (8)	0.0535 (3)	
O3	0.2481 (3)	0.12887 (17)	0.50560 (9)	0.0830 (5)	
O4	−0.2254 (3)	0.38059 (15)	0.60001 (9)	0.0660 (4)	
O5	−0.1328 (2)	0.27460 (13)	0.42790 (8)	0.0557 (3)	
N1	0.1791 (2)	0.24475 (13)	0.68396 (9)	0.0422 (3)	
C1	0.3864 (3)	0.23438 (16)	0.86482 (11)	0.0420 (3)	
C2	0.5692 (3)	0.1711 (2)	0.92768 (13)	0.0551 (4)	
C3	0.7340 (4)	0.0645 (2)	0.87854 (15)	0.0602 (4)	
C4	0.7185 (3)	0.01962 (18)	0.76469 (14)	0.0533 (4)	
C5	0.5376 (3)	0.07796 (17)	0.70010 (12)	0.0463 (3)	
C6	0.3673 (3)	0.18576 (15)	0.74833 (10)	0.0385 (3)	
C7	0.2187 (3)	0.35013 (17)	0.92252 (11)	0.0456 (3)	
C8	0.1306 (3)	0.21176 (16)	0.57239 (10)	0.0451 (3)	
C9	−0.1000 (3)	0.29967 (16)	0.53589 (11)	0.0446 (3)	
C10	−0.3469 (4)	0.3514 (2)	0.38191 (15)	0.0622 (5)	
C11	−0.3098 (6)	0.3297 (3)	0.26382 (17)	0.0828 (7)	
H1	0.139 (5)	0.455 (3)	1.061 (2)	0.099 (8)*	
H1A	0.065 (4)	0.311 (2)	0.7222 (17)	0.070 (6)*	
H2	0.581 (4)	0.206 (2)	1.0049 (19)	0.078 (6)*	
H3	0.856 (4)	0.025 (2)	0.9243 (18)	0.081 (6)*	
H4	0.836 (4)	−0.0499 (19)	0.7304 (16)	0.058 (5)*	
H5	0.532 (4)	0.046 (2)	0.6220 (16)	0.054 (4)*	
H10A	−0.321 (4)	0.452 (2)	0.4258 (18)	0.077 (6)*	
H10B	−0.517 (4)	0.307 (2)	0.3905 (17)	0.075 (6)*	
H11A	−0.121 (6)	0.375 (3)	0.260 (2)	0.114 (9)*	
H11B	−0.326 (5)	0.229 (3)	0.224 (2)	0.109 (9)*	
H11C	−0.457 (5)	0.377 (3)	0.230 (2)	0.096 (7)*	

### Atomic displacement parameters (Å^2^)

**Table:**

	*U*^11^	*U*^22^	*U*^33^	*U*^12^	*U*^13^	*U*^23^
O1	0.0815 (8)	0.0954 (9)	0.0244 (5)	0.0438 (7)	0.0077 (5)	0.0081 (5)
O2	0.0659 (7)	0.0677 (7)	0.0261 (5)	0.0287 (5)	0.0102 (4)	0.0077 (4)
O3	0.1083 (10)	0.1073 (10)	0.0320 (6)	0.0657 (9)	0.0163 (6)	0.0079 (6)
O4	0.0726 (8)	0.0869 (9)	0.0377 (6)	0.0387 (6)	0.0092 (5)	0.0122 (6)
O5	0.0683 (7)	0.0699 (7)	0.0289 (5)	0.0181 (5)	0.0034 (5)	0.0144 (5)
N1	0.0479 (6)	0.0527 (7)	0.0251 (5)	0.0165 (5)	0.0087 (4)	0.0070 (5)
C1	0.0421 (7)	0.0520 (8)	0.0306 (7)	0.0093 (6)	0.0066 (5)	0.0089 (6)
C2	0.0592 (9)	0.0720 (10)	0.0338 (7)	0.0177 (7)	0.0036 (6)	0.0150 (7)
C3	0.0601 (10)	0.0710 (10)	0.0518 (9)	0.0246 (8)	0.0020 (7)	0.0218 (8)
C4	0.0539 (9)	0.0549 (9)	0.0525 (9)	0.0201 (7)	0.0124 (7)	0.0136 (7)
C5	0.0509 (8)	0.0510 (8)	0.0364 (7)	0.0127 (6)	0.0110 (6)	0.0089 (6)
C6	0.0401 (6)	0.0452 (7)	0.0299 (6)	0.0072 (5)	0.0073 (5)	0.0093 (5)
C7	0.0493 (8)	0.0588 (8)	0.0261 (6)	0.0117 (6)	0.0062 (5)	0.0072 (6)
C8	0.0543 (8)	0.0521 (8)	0.0269 (6)	0.0143 (6)	0.0090 (5)	0.0060 (5)
C9	0.0510 (8)	0.0505 (8)	0.0294 (6)	0.0078 (6)	0.0043 (5)	0.0076 (6)
C10	0.0689 (11)	0.0728 (12)	0.0470 (9)	0.0097 (9)	−0.0051 (8)	0.0260 (8)
C11	0.1075 (18)	0.0977 (17)	0.0459 (10)	0.0012 (14)	−0.0099 (10)	0.0354 (11)

### Geometric parameters (Å, º)

**Table:**

O1—C7	1.3116 (17)	C2—H2	0.94 (2)
O1—H1	0.96 (3)	C3—C4	1.377 (2)
O2—C7	1.2258 (17)	C3—H3	0.94 (2)
O3—C8	1.2007 (18)	C4—C5	1.371 (2)
O4—C9	1.1963 (18)	C4—H4	0.939 (18)
O5—C9	1.3122 (16)	C5—C6	1.3955 (19)
O5—C10	1.456 (2)	C5—H5	0.948 (19)
N1—C8	1.3493 (17)	C8—C9	1.532 (2)
N1—C6	1.3944 (18)	C10—C11	1.491 (3)
N1—H1A	0.93 (2)	C10—H10A	0.95 (2)
C1—C2	1.392 (2)	C10—H10B	0.95 (2)
C1—C6	1.4084 (18)	C11—H11A	1.01 (3)
C1—C7	1.474 (2)	C11—H11B	0.93 (3)
C2—C3	1.374 (2)	C11—H11C	0.98 (3)
			
C7—O1—H1	108.9 (15)	C5—C6—C1	118.68 (13)
C9—O5—C10	116.37 (13)	O2—C7—O1	121.64 (13)
C8—N1—C6	128.24 (12)	O2—C7—C1	124.02 (12)
C8—N1—H1A	115.5 (12)	O1—C7—C1	114.35 (12)
C6—N1—H1A	116.1 (12)	O3—C8—N1	127.84 (14)
C2—C1—C6	119.03 (13)	O3—C8—C9	121.08 (13)
C2—C1—C7	118.80 (12)	N1—C8—C9	111.06 (11)
C6—C1—C7	122.16 (12)	O4—C9—O5	126.54 (14)
C3—C2—C1	121.37 (14)	O4—C9—C8	122.79 (12)
C3—C2—H2	121.1 (13)	O5—C9—C8	110.66 (12)
C1—C2—H2	117.5 (13)	O5—C10—C11	106.64 (18)
C2—C3—C4	119.29 (15)	O5—C10—H10A	106.7 (13)
C2—C3—H3	118.4 (13)	C11—C10—H10A	113.3 (13)
C4—C3—H3	122.3 (13)	O5—C10—H10B	105.1 (12)
C5—C4—C3	120.89 (14)	C11—C10—H10B	113.6 (13)
C5—C4—H4	119.0 (11)	H10A—C10—H10B	110.7 (18)
C3—C4—H4	120.1 (11)	C10—C11—H11A	109.9 (15)
C4—C5—C6	120.72 (14)	C10—C11—H11B	111.3 (16)
C4—C5—H5	119.4 (11)	H11A—C11—H11B	109 (2)
C6—C5—H5	119.9 (11)	C10—C11—H11C	108.4 (15)
N1—C6—C5	121.55 (12)	H11A—C11—H11C	110 (2)
N1—C6—C1	119.77 (11)	H11B—C11—H11C	108 (2)
			
C6—C1—C2—C3	1.2 (2)	C2—C1—C7—O2	178.82 (14)
C7—C1—C2—C3	−178.93 (16)	C6—C1—C7—O2	−1.3 (2)
C1—C2—C3—C4	0.2 (3)	C2—C1—C7—O1	−1.2 (2)
C2—C3—C4—C5	−1.2 (3)	C6—C1—C7—O1	178.69 (13)
C3—C4—C5—C6	0.8 (3)	C6—N1—C8—O3	−3.3 (3)
C8—N1—C6—C5	−0.1 (2)	C6—N1—C8—C9	178.43 (13)
C8—N1—C6—C1	−179.63 (13)	C10—O5—C9—O4	−1.1 (2)
C4—C5—C6—N1	−178.99 (14)	C10—O5—C9—C8	179.88 (14)
C4—C5—C6—C1	0.5 (2)	O3—C8—C9—O4	178.26 (18)
C2—C1—C6—N1	178.03 (13)	N1—C8—C9—O4	−3.3 (2)
C7—C1—C6—N1	−1.9 (2)	O3—C8—C9—O5	−2.7 (2)
C2—C1—C6—C5	−1.5 (2)	N1—C8—C9—O5	175.70 (12)
C7—C1—C6—C5	178.62 (13)	C9—O5—C10—C11	169.35 (16)

### Hydrogen-bond geometry (Å, º)

**Table:**

*D*—H···*A*	*D*—H	H···*A*	*D*···*A*	*D*—H···*A*
O1—H1···O2^i^	0.96 (3)	1.67 (3)	2.6274 (16)	176 (2)
N1—H1*A*···O2	0.93 (2)	1.92 (2)	2.6574 (15)	135.1 (16)
C5—H5···O3	0.948 (19)	2.230 (18)	2.866 (2)	123.7 (14)
C5—H5···O3^ii^	0.948 (19)	2.36 (2)	3.1107 (19)	135.9 (14)

Symmetry codes: (i) −*x*, −*y*+1, −*z*+2; (ii) −*x*+1, −*y*, −*z*+1.
